# Admission prioritization of heart failure patients with multiple comorbidities

**DOI:** 10.3389/fdgth.2024.1379336

**Published:** 2024-07-02

**Authors:** Rahul Awasthy, Meetu Malhotra, Michael L. Seavers, Mark Newman

**Affiliations:** Data Science, Harrisburg University of Science and Technology, Harrisburg, PA, United States

**Keywords:** triage, machine learning, prioritization, heart failure, HCUP, PhysioNet, GCS, CCI

## Abstract

The primary objective of this study was to enhance the operational efficiency of the current healthcare system by proposing a quicker and more effective approach for healthcare providers to deliver services to individuals facing acute heart failure (HF) and concurrent medical conditions. The aim was to support healthcare staff in providing urgent services more efficiently by developing an automated decision-support Patient Prioritization (PP) Tool that utilizes a tailored machine learning (ML) model to prioritize HF patients with chronic heart conditions and concurrent comorbidities during Urgent Care Unit admission. The study applies key ML models to the PhysioNet dataset, encompassing hospital admissions and mortality records of heart failure patients at Zigong Fourth People's Hospital in Sichuan, China, between 2016 and 2019. In addition, the model outcomes for the PhysioNet dataset are compared with the Healthcare Cost and Utilization Project (HCUP) Maryland (MD) State Inpatient Data (SID) for 2014, a secondary dataset containing heart failure patients, to assess the generalizability of results across diverse healthcare settings and patient demographics. The ML models in this project demonstrate efficiencies surpassing 97.8% and specificities exceeding 95% in identifying HF patients at a higher risk and ranking them based on their mortality risk level. Utilizing this machine learning for the PP approach underscores risk assessment, supporting healthcare professionals in managing HF patients more effectively and allocating resources to those in immediate need, whether in hospital or telehealth settings.

## 1 Introduction

### 1.1 Background

With the increase in heart patients worldwide and the limitations of care resources, it has become imperative to take advantage of the patient prioritization (PP) method, especially if it is machine learning (ML)-driven [or artificial intelligence (AI)] under the supervision of experienced healthcare staff (1). Prioritizing heart failure (HF) patients based on their mortality risk at admission to an Urgent Care Unit helps allocate limited and focused resources, such as beds, ambulances, specialist doctors and nurses, and diagnostic machines, effectively to HF patients. This process leads to improved precision care, reduced response times, and increased chances of survival and recovery for HF patients.

### 1.2 Importance

Patient classification and prioritization (triage) (2) are essential for providing quick and timely care services to virtual and in-facility patients with HF (3). PP entails arranging patient cases or referrals in a specific sequence, considering various criteria aimed at reducing patient wait times (4), enhancing healthcare accessibility, and optimizing operational efficiencies. Prioritization is a crucial competency for healthcare personnel because it ensures that patients are addressed in an order that maximizes overall patient wellbeing, safety, and health (5). Notably, most of the current machine learning models (6) for automated patient prioritization in HF do not incorporate factors (both medical condition at hand and chances of mortality), such as comorbidities, age, patient prognosis factors, diagnostic/clinical outcomes, readmission history, or medication data, to assess the criticality of patients’ healthcare needs effectively (7).

The critical point to understand here is that heart failure or cardiovascular disease (CVD) [impaired myocardial perfusion (8) and inflammation] is not an isolated condition and gets severely impacted by key comorbidities (9), i.e., chronic kidney disease (CKD), liver disease, renal failure, chronic respiratory conditions, depression, cancers, and diabetes (10). Another critical data item missed in the current automated PP model is the usage of the correct set of medicines (11) that can reduce the impact of mortality and readmissions for patients with HF (12) and improve adherence to medication and self-care (13). This research builds on the current gaps and challenges to generate an automated PP model that provides a robust HF patient prioritization decision tool for patients with acute heart failure and morbidities.

### 1.3 Goals of investigation

Patient classification and prioritization ML models work on a three-tier architecture: tier 1, collecting patient data; tier 2, patient data storage (cloud/on-premises) for running ML models; and tier 3, healthcare applications for implementing ML outcomes. This research focuses on tier 3 for creating AI/ML models with the success criteria below.

First, generate the ranking (in order of their high- to low-risk levels) of patients with HF based on the fundamental health parameters using a machine learning (or AI) model (14). Second, identify key health assessment parameters (15) for HF patients to be assessed by healthcare staff at the time of hospital/care unit admission. Finally, assess how HF patient ranking and key parameter outcomes improve current gestalt predictions by nurses or doctors, either in isolation or in conjunction with clinical evaluation.

## 2 Materials and methods

This initiative is associated with generating machine learning models to predict patient mortality (worsening of future health conditions) based on HF patients’ health conditions, readmission, medicinal usage, and other diagnostic/clinical factors captured during hospital/care facility admission (16). Overall, the model uses available information, such as patient prognosis factor (physical), diagnosis and clinical conditions, medicine history, comorbidity details, age sensitivity (17), and chances of mortality, to generate an effective classification and prioritization of patients for immediate care. The Institutional Review Board (IRB) of Harrisburg University of Science and Technology institution exempted the research from ethical review (Waived Ethical Review number: IRB 20231029).

### 2.1 Dataset

The primary dataset, PhysioNet, aims to facilitate epidemiological studies of heart failure and is vital in providing optimal care to reduce patient populations and healthcare system differences between China and other countries (18, 19). The dataset includes 168 variables for 2,008 patients with heart failure (at first-time care admission), and close to 43 variables (refer to Tables 1, 2) were identified for research on patient admission. Data on subsequent hospital admissions and mortality were obtained at a mandatory follow-up visit at 28 days, 3 months, and 6 months. Medications administered during hospitalization are recorded in this database. This dataset’s primary drug categories were diuretics, inotropes, and vasodilators. The diuretics drug included furosemide, torsemide, and spironolactone. The inotrope drugs included deslanoside, dobutamine, digoxin, isoprenaline, and milrinone. The vasodilator drug included isosorbide mononitrate and nitroglycerin (20).

**Table 1:** Dependent variable—admission.

Variable	Description	Data type
Death within 6 months	HF patient death within 6 months	Binary

The table shows the dependent variable used in the Admission Prioritization model.

**Table 2:** Independent variables—admission.

Variables	Description	Data type
Inpatient number	Unique patient ID	Continuous
Admission way	Possible ways of admission are emergency vs. non-emergency	Binary
Gender	Gender: Male, Female	Binary
Body temperature	Body temperature in °C	Continuous
Pulse	Pulse rate (beats/min)	Continuous
Respiration	Respiratory rate (breaths/min)	Continuous
Systolic blood pressure	Systolic blood pressure (mmHg)	Continuous
Diastolic blood pressure	Diastolic blood pressure (mmHg)	Continuous
Mitral valve (EMS)	Maximum velocity of the mitral valve E wave (m/s)	Continuous
Mitral valve (AMS)	Maximum velocity of the mitral valve A wave (m/s)	Continuous
BMI	BMI (kg/m^2^)	Continuous
Type of heart failure	Type of heart failure (left, right, both)	Binary
Congestive heart failure	Congestive heart failure (number for first-time HF patients)	Binary
Dementia	Dementia indicator	Binary
Chronic obstructive disease	Chronic obstructive pulmonary disease	Binary
Peptic ulcer disease	Peptic ulcer disease	Binary
Diabetes	Diabetes	Binary
Moderate to severe chronic kidney disease	Moderate to severe chronic kidney disease with glomerular filtration rate <60 ml/min	Binary
Solid tumor	A solid tumor	Binary
Liver disease	Liver disease	Binary
AIDS	AIDS	Binary
CCI score	Charlson comorbidity index score	Continuous
LVEF	Left ventricular ejection fraction	Continuous
Consciousness	Consciousness	Binary
Eye-opening	Eye-opening	Continuous
Verbal response	Verbal response	Continuous
Movement	Movement	Continuous
Respiratory support	Use of either invasive or non-invasive mechanical ventilation	Binary
Oxygen inhalation	Oxygen inhalation	Binary
Acute renal failure	The presence of acute kidney injury is defined as an increase in serum creatinine	Binary
Readmission within 28 days	Readmission within 28 days	Binary
Readmission within 3 months	Readmission within 3 months	Binary
Readmission within 6 months	Readmission within 6 months	Binary
Oxygen saturation	Oxygen saturation (%)	Continuous
Age category	The age is categorized into decades	Continuous
Diuretics	A diuretic is any substance that promotes diuresis, increases the production of urine, and reduces blood pressure	Binary
Inotropic	Inotropic agents are a group of medicines that affect the contraction of the heart muscle	Binary
Vasodilators	Vasodilators are medicines that dilate (widen) blood vessels, allowing blood to flow more easily through	Binary

The table shows the set of independent variables used in the Admission Prioritization model.

The research further verified the results of patient prioritization outcomes from the PhysioNet dataset with the secondary dataset Healthcare Cost and Utilization Project (HCUP) Maryland (MD) State Inpatient Data (SID) for 2014 with heart failure patients to identify the applicability of results in more comprehensive healthcare settings across geographies and different patient settings (21, 22). This database contains close to 48,000 records for HF patients admitted to MD state hospitals in 2014.

### 2.2 Patient prioritization architecture

**Figure 1:**
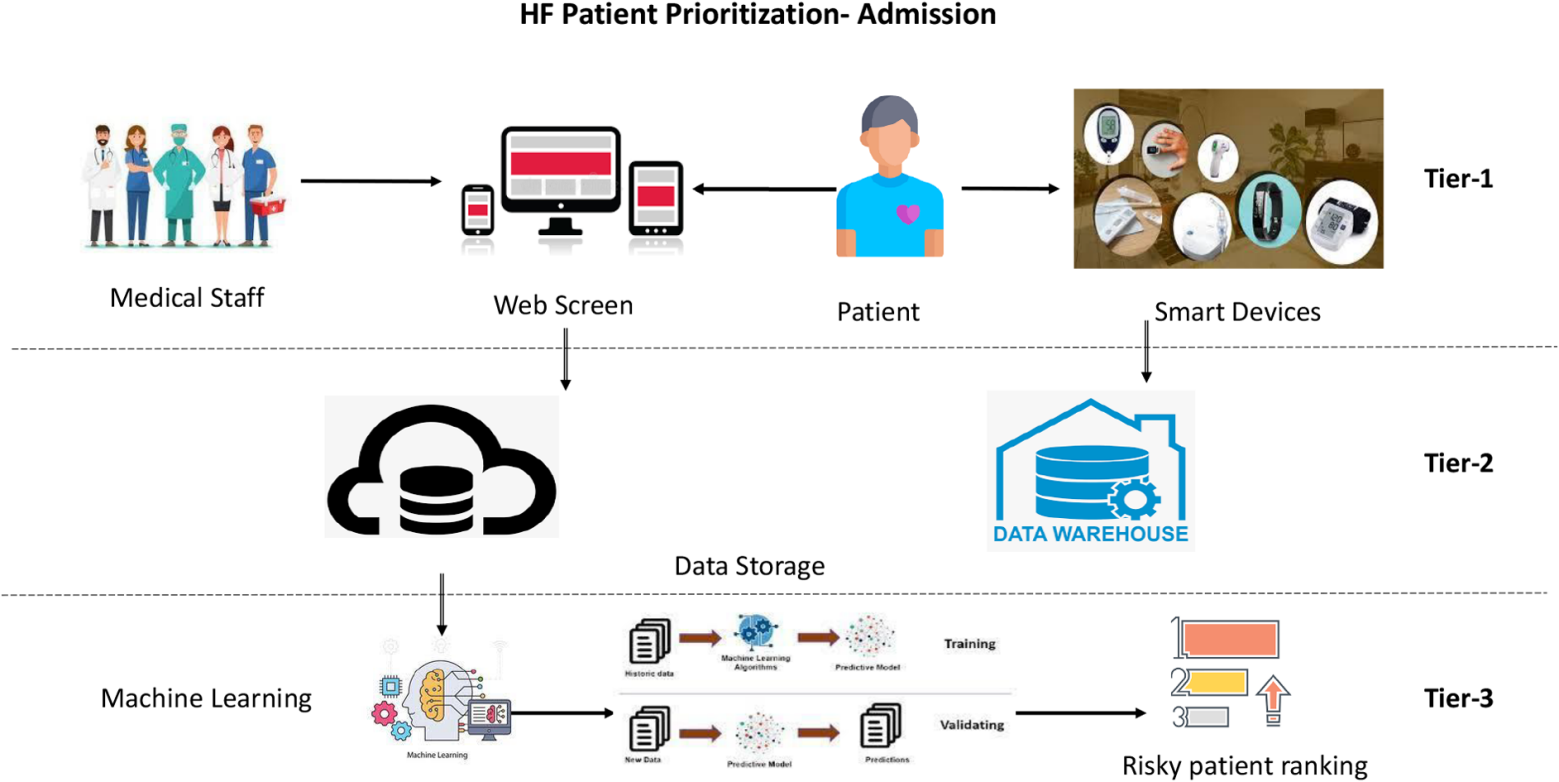
Three-tiered prioritization approach for HF patients during the admissions process.

This research generates a real-time patient monitoring and prioritization model (refer to Figure 1) that obtains inputs on multiple heterogeneous clinical and non-clinical parameters for HF patients entered manually or pulled through wearable devices/user interface software.

#### 2.2.1 Identification of clinical and observational parameters (tier 1)

The user interface can capture clinical, non-clinical, biochemical, physical, and observational parameters for HF patients in hospital emergencies (23), ambulances, telehealth devices, or health monitoring devices/transmitters. This data-capturing process applies to HF patients [or severe CVD patients] with pre-existing relationships with the healthcare system/hospital chain or to newer patients who have an intervention with the healthcare system to provide/insert patient data.

#### 2.2.2 Identification of online data collection systems (tier 2)

Online data collection includes systems that send clinical data to server-side machines to run real-time ML models. These data collection systems could be mobile phones, laptops, clinical data collection machines, or any other method to put the captured data for the HF patients into a data store, server, or cloud.

#### 2.2.3 Generation of real-time machine learning models (tier 3)

This research focuses on tier 3 to create a machine learning model for HF patient classification and prioritization. Below are the steps followed in this tier:

• Data imputation techniques: K-nearest neighbor (KNN) imputation is an analytics technique used to replace missing data with a substitute value to retain most of the data/information of the dataset as KNN impute provides a more robust and sensitive method for missing value estimation.
• Classification and prioritization: support vector machine (SVM), logistic regression, decision tree, random forest, and linear regression ML models are used to classify (risk buckets) and prioritize (ranking high to low risk) the HF patients and the one with the best outcome is selected (shortlisted) for the final version.
• Causal inference model: the causal inference mathematical model (linear regression/Bayesian/binomial) is created in this research to incorporate the idea of multiple causalities. This work helps understand the impact of various admission-related independent variables on HF patient mortality and explains which independent variable impacts mortality more/less than other variables.
• Ranking of critical HF patients: based on the ranking created for the urgent (high-risk) and immediate care of HF patients in Urgent Care Admissions, telemedicine (24), ambulance, or any other option for care scenarios, healthcare services, resources, doctors, nurses, and infrastructure can be assigned to a patient with the immediate needs.
• Verification: in this step, the functioning of the HF patient prioritization model generated with the PhysioNet dataset is verified by running the models on the heart failure dataset from patient admission/hospitalization for HCUP Maryland state 2014 to check the uniformity of model results (25).
• Validation: based on the outcome of the predictive model, regression coefficients/weights are identified for all the independent variables. These weights or risk factors are compared to past research work for the Canadian Triage and Acuity Scale (CTAS) to verify how the current model performs concerning the past patient prioritization models (26, 27).

## 3 Results

### 3.1 Baseline characteristics (data imputation)

The KNN data imputation technique is applied to impute the missing data for columns electrocardiogram (ECG)-EMS (E wave), ECG-AMS (A wave), oxygen saturation, peptic ulcer, CKD, left ventricular ejection fraction (LVEF), Charlson comorbidity index (CCI) score, return to emergency department (ED) (28), and liver disease. HF patient health data are divided into five key segments [classification/prognosis parameters, diagnostic parameters (clinical and non-clinical), patient’s medical history, patient's readmission data, and comorbidities] to compare the data for patients who died within 6 months and those alive after 6 months of hospital admission.

### 3.2 Exploratory data analysis (classification)

#### 3.2.1 First bucket/classification

Patient classification/prognosis factors include the following: the Glasgow Coma Scale/Score (GCS), which determines the patient's condition through verbal, eye-opening, and motor response (movements) (refer to Supplementary Figure S1); New York Heart Association (NYHA) classification, which divides the stages of heart failure into four stages based on physical symptoms (refer to Supplementary Figure S2); Killip classification, which is based on physical examination to identify the development of heart failure to predict (29) and stratify their mortality risk (30) from class 1 to class IV from no congestion stage to cardiogenic shock (31) (refer to Supplementary Figure S3); and CCI, which generates a score by summing the assigned weights of all comorbid conditions (refer to Supplementary Figure S4). Patients with lower GCS averaging 12 died within 6 months compared to GCS of 15. Similarly, NYHA grades 4 and 3 died more often than NYHA grades 1 and 2. In addition, 27% of patients with a Killip grade of 4 died within 6 months. Patients who died had a higher average CCI score of 1.96 compared to 1.85 for those who lived after 6 months (refer to Supplementary Table S1).

#### 3.2.2 Second bucket/classification

The diagnostic and clinical data of HF patients include the requirement for respiratory support, blood pressure of the patient, mitral valve opening values [ECG related (32)], LVEF [echocardiogram (ECHO) output], and body mass index (BMI). Key observations are that 9% of patients requiring respiratory support died within 6 months; patients with an average mitral valve opening value of 0.93 died compared to those with a mitral opening average near 4.2–4.4 (refer to Supplementary Table S2).

#### 3.2.3 Third bucket/classification

The medicine history covers the intake of three critical medicines: diuretics (water pills), inotropics (change heart contractions), and vasodilators (dilate vessels). Only 2%–3% of patients taking these medications died within 6 months of admission (97%–98% lived for 6 months while consuming the drug), showing medicine intake as a significant independent variable (refer to Supplementary Table S3).

#### 3.2.4 Fourth bucket/classification

Hospital readmission data of patients identify that patients who were readmitted 6 months after their first admission did not die within 6 months and continued living (refer to Supplementary Table S4).

#### 3.2.5 Fifth bucket/classification

The patient's comorbidity conditions include liver disease, CKD, renal failure, diabetes, dementia, and chronic obstructive pulmonary disease (COPD). Of the patients who had liver disease, 11% died within 6 months, 29% of patients with renal failure died within 6 months, 5% of patients with CKD died within 6 months (approximately 24% of the total population had CKD), and 3% of patients with diabetes died within 6 months (23% of the total population had diabetes) (refer to Supplementary Table S5).

### 3.3 Feature selection (classification and prioritization)

The ultimate choice of input variables for machine learning models, comprising 39 variables, is determined through the utilization of the chi-square test for categorical variables, the analysis of variance (ANOVA) test for numerical data, and the consideration of correlation factors (see Supplementary Figures S5–S7). In addition, prominent ML classification models such as logistic regression, SVM, and random forest were applied to the entire PhysioNet research database using the initial set of 39 variables (cross-validated with the HCUP dataset) for patients with HF. The aim of this process was to identify and narrow down to 25 variables that demonstrated enhanced model efficiencies, improved cross-validation scores, sensitivity, specificity, and area under the curve (AUC).

### 3.4 Machine learning analysis (classification and prioritization)

The ML models used in this research belong to two key categories: interpretable models, such as logistic regression, decision tree, and linear regression (Bayesian); and opaque models, such as SVM and random forest. Initially, all the ML models were run on a complete set of 39 variables. However, based on the outcome of crucial machine learning models, exploratory analysis, and basic statistical techniques, critical variables were shortlisted (close to 25) for HF patient prioritization at admission.

**Figure 2:**
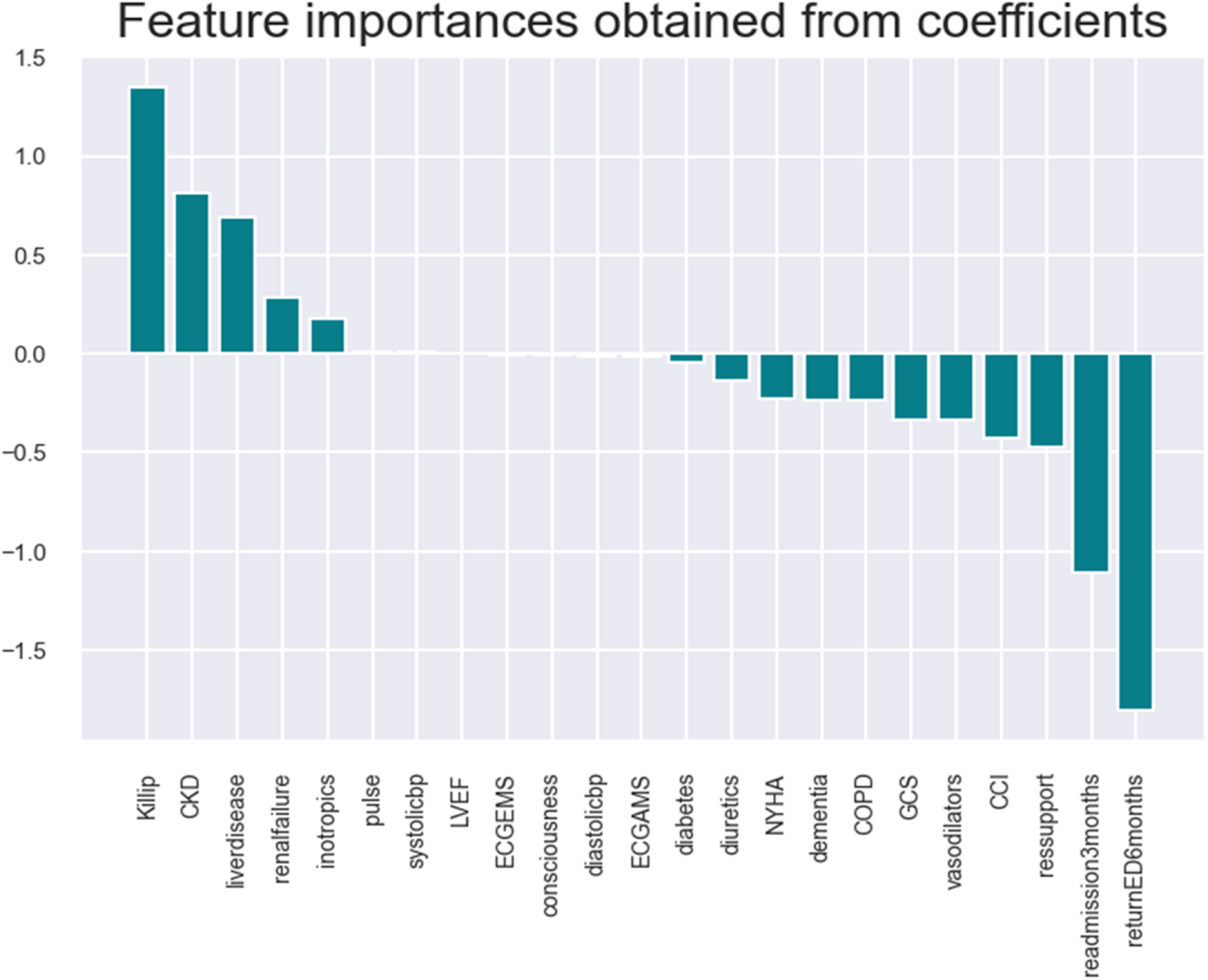
A logistic regression model with shortlisted critical variables for HF patients.

**Figure 3:**
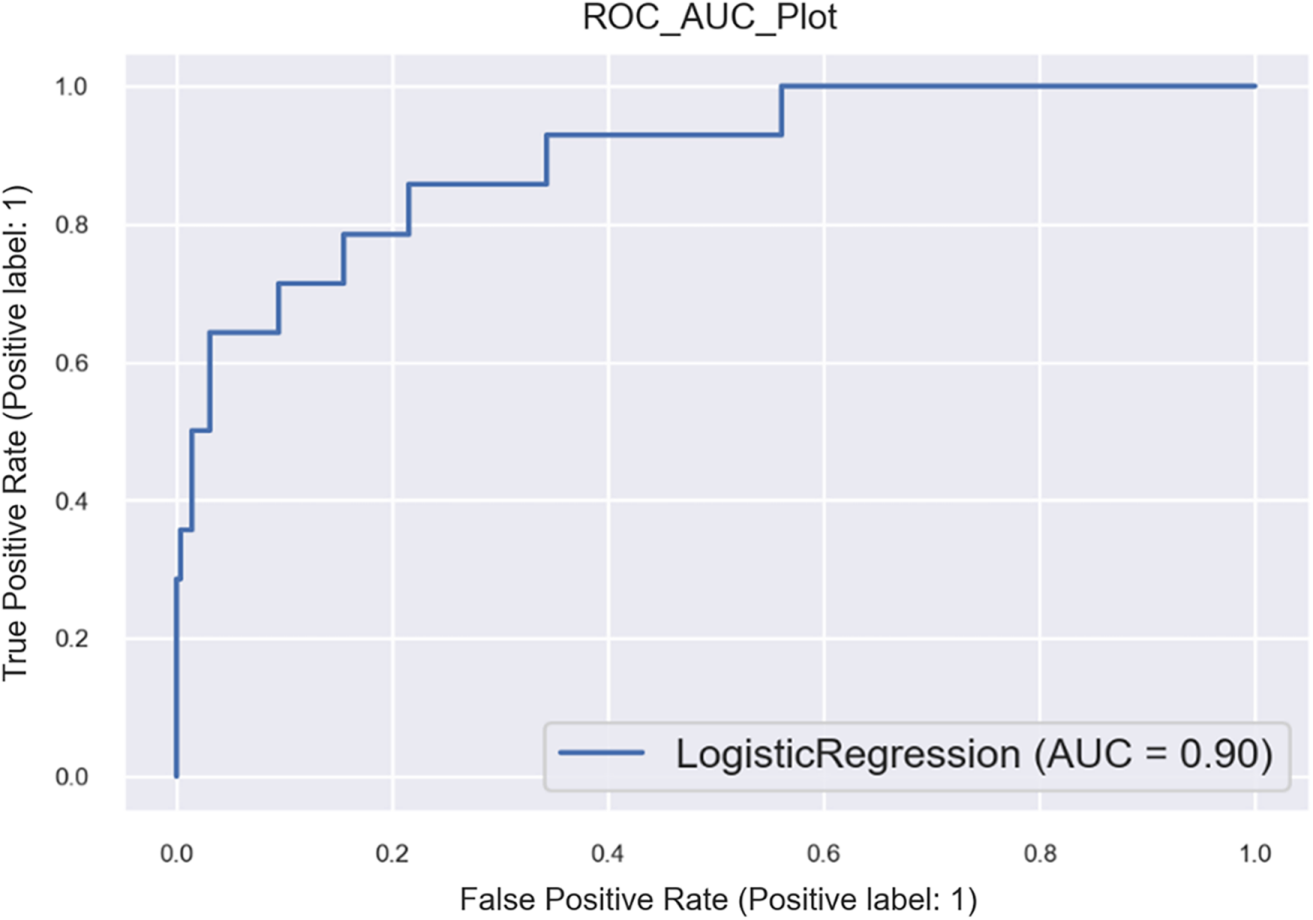
AUC curve of a logistic regression model with shortlisted variables for HF patients.

The outcome of five key machine learning techniques with 25 variables (refer to Table 3) used in the research are below:

• Logistic: model accuracy of 98.18%, cross-validation of 84.2%, and receiver operating characteristic (ROC) score of 68% (refer to Figures 2, 3 and Supplementary Figures S9, S10).

**Table 3:** Comparison table for machine learning models—admissions.

Model	Model accuracy (%)	Cross-validation (%)	ROC score (%)	Sensitivity (%)	Specificity (%)
Logistic	98.18	84.20	68	27	99.70
Support vector	98	83	50	16.20	99.70
Random forest	98.34	83.60	64.29	29	99.80
Decision tree	98.01	71	64.12	29	99.80
Bayesian regression	80–94	NA	NA	NA	NA

The table compares the performances of ML models applied in the research.

The logistic model identifies the following key health parameters to be relevant at admission for patients with HF: classification/prognosis factor (Killip/CCI/GCS), comorbidity (liver disease, CKD, etc.), medicine intake (inotropic, vasodilator, and diuretics),. readmission (at 3/6 months), and diagnostic/demographic/clinical (blood pressure, pulse, mitral valve/ECG, and respiratory support).

**Figure 4:**
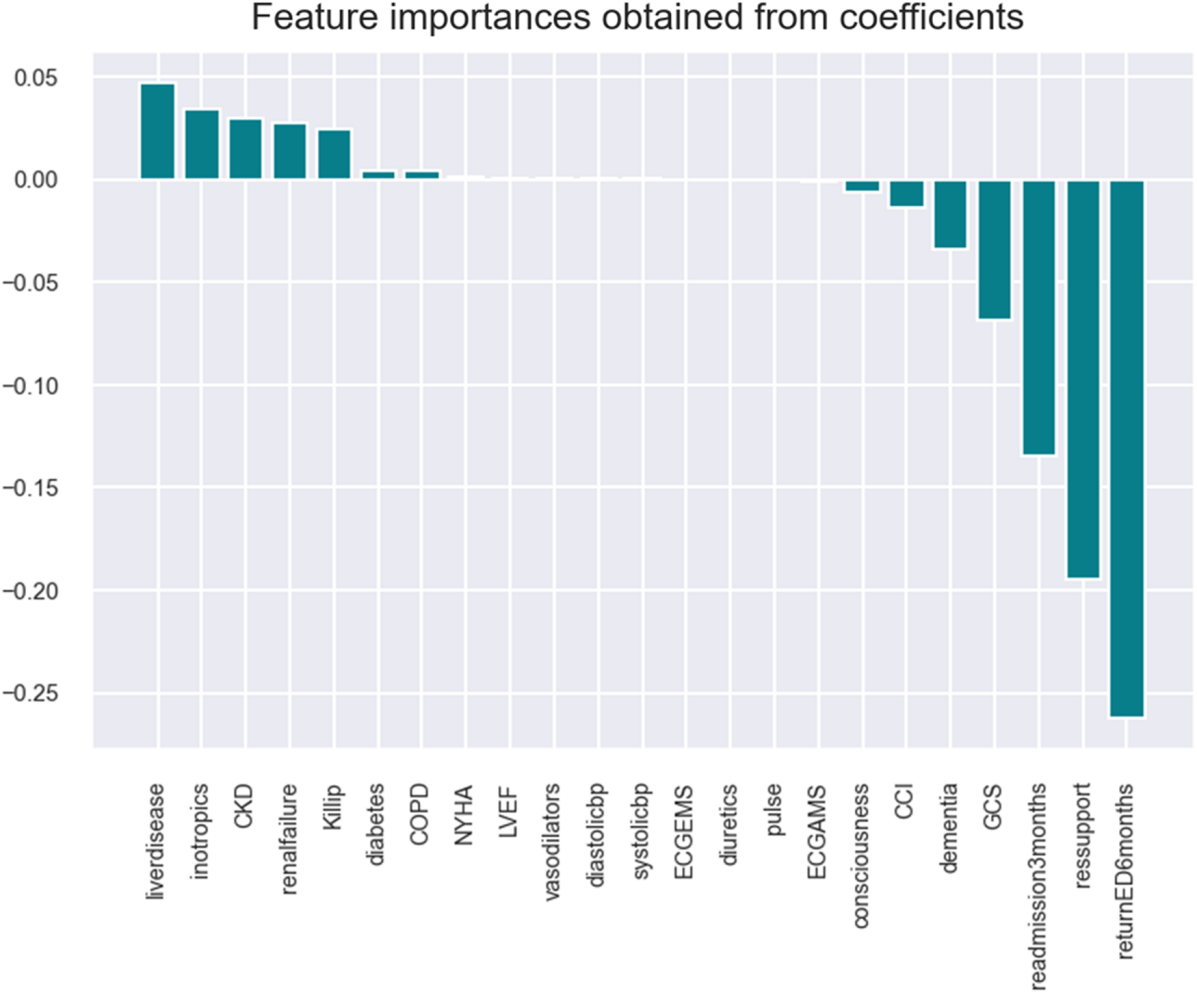
SVM model with shortlisted critical variables for HF patients.

• Support vector: model accuracy of 98%, cross-validation of 83%, and ROC score of 50% (refer to Figure 4 and Supplementary Figures S11, S12).

The SVM model identifies the following key health parameters to be relevant at admission for patients with HF: classification factor (Killip, GCS, and CCI), comorbidity (liver disease, CKD, COPD, dementia, renal failure, and diabetes), medicine intake (inotropic), readmission (return to ED and readmission at 3 months), and diagnostic (mitral valve/ECG and respiratory support).

• Random forest: the random forest classifier (refer to Supplementary Figures S13, S14) is a supervised learning algorithm that can be used for regression and classification problems. It shows model accuracy of 98.34%, cross-validation score of 83.60%, and ROC score of 64.29%.

The random forest model identifies the following key health parameters to be relevant at admission for patients with HF: classification factor (consciousness, Killip, GCS, and CCI), comorbidity (renal failure, CKD, liver disease), medicine intake (vasodilators and inotropic), readmission (return to ED), and diagnostic (blood pressure, pulse, mitral valve/ECG, LVEF/ECHO, and respiratory support).

• Decision tree: model accuracy of 98.01%, cross-validation score of 71%, and ROC score of 64.12% (refer to Supplementary Figure S8).

The decision tree model identifies the following key health parameters to be relevant at admission for patients with HF: HF patient's condition parameter (such as GCS and CCI), diagnostic variables [myocardial infraction (33), pulse, body temperature, sex, and age], and comorbidity data (Killip and non-cardiac variables).

The outcome of exploratory, statistical, and ML analyses shows that the classification, diagnostic, medicine intake, readmission, and comorbidity buckets encompass all the essential health parameters of HF patients to apply emergency/urgent interventions (refer to Supplementary Tables S6–S8).

• Linear regression (causal inference/binomial/Bayesian regression): five buckets identified in the exploratory analysis to ML modeling were used in Bayesian regression models (descriptive analytics) to check the importance of HF patients’ admission parameters and found to be aligned with earlier analysis. Model accuracy is in the range of 80%–94% (based on the variables in classification buckets).

The following are the key health parameters found through the Bayesian analysis/model: classification parameters (GCS, NYHA), diagnostic variables (blood pressure and ECG), medicinal (diuretics), readmission variables (admission within 3 months), and comorbidity (liver disease, CKD) (refer to Supplementary Table S9).

Refer to Table 4 for comparing seven essential health parameters for HF patients (shortlisted by ML models) and a visualization of the feature importance of the different variables in the various models.

**Table 4:** Comparison table for key health parameters for patients with heart failure—admissions.

Model	Feature 1	Feature 2	Feature 3	Feature 4	Feature 5	Feature 6	Feature 7
Logistic	Killip grade	Return to emergency	Readmission	CKD	Liver disease	Type of HF	GCS
Support vector	GCS	Return to emergency	Respiration support	Readmission	AIDS	Liver disease	CKD
Random forest	GCS	Killip grade	BP	Pulse	Oxygen saturation	CCI	LVEF
Decision tree	GCS	Pulse	BP	CCI	LVEF	Gender	Killip grade
Bayesian regression	Return to emergency	Diuretics intake	Readmission	Liver Disease	CKD	Vasodilator intake	COPD

The table shows the seven key features identified by the ML models applied in the research.

### 3.5 ACC vs. sensitivity vs. specificity

Below are the critical parameters of the three best ML models per their outcomes (with 25 shortlisted variables). These ML models identify the key health parameters to be considered by health staff for HF patient admission and generate a ranking of higher-risk patients with HF based on chances of mortality. Refer to Table 3 above for ACC, Sensitivity and Specificity details.

Model accuracy (ACC): the significance of model accuracy lies in its capacity to assess a model’s proficiency in processing, comprehending, and predicting the outcomes of patients with HF. Logistic: 98.18%; SVM: 98%; random forest: 98.34%; decision tree: 97.84%.

Sensitivity: the sensitivity value assessed the ability of the ML model to detect positive instances. This study’s sensitivity value applies to identifying correct patients at higher risk of HF. Logistic: 27%; SVM: 16.2%; random forest: 29%; decision tree: 29%.

Specificity: the specificity value assessed the ability of the ML model to detect true negative instances. This study's specificity value applies to identifying HF patients who are not at high risk. Logistic: 99.7%; SVM: 99.7%; random forest: 99.8%; decision tree: 99.8%.

The machine learning models used in this research are calibrated to prioritize higher specificity, aiming to minimize the risk of classifying heart failure (HF) patients with lower risk as having a higher risk of HF mortality. This outcome encourages a targeted approach toward identifying and addressing the needs of HF patients at higher risk. Refer to (34) with similar type work focussed on higher specificity and moderate sensitivity accepted by The National Institute of Health (USA).

### 3.6 Verification with HCUP data

Common variable types/buckets from the PhysioNet (Asian dataset) HF dataset are found to be in sync with HCUP (US dataset) data (refer to Table 5). The similarity of HF patients’ health variables (at admission) shows that the results from the primary research study can be applied across geographies and healthcare settings. The key parameter buckets found from the exploratory, statistical (refer to Supplementary Figure S15), and ML modeling (refer to Supplementary Figures S16–S18) of the HCUP dataset are comorbidities (such as diabetes), diagnostic (such as ECG), patient classification (such as CCI), medicine/drug intake, and readmission to hospital (refer to Supplementary Table S10).

**Table 5:** Comparison table of key variables PhysioNet vs. HCUP datasets—admissions.

PhysioNet dataset	HCUP data (Maryland State, USA)
Demographic variables (BMI, age, gender)	Demographic (obesity)
Clinical (respiratory support, BP, pulse)	Clinical (therapies, respiration/physical)
Comorbidities(liver disease, CKD, renal failure, diabetes, dementia, COPD, CHF)	Comorbidities, such as diabetes, tumor/cancer, and depression (close to dementia)
Classification factors (GCS, NYHA, Killip, CCI)	Patient classification variables (chronic illness, CCI)
Diagnostic (LVEF, mitral valve/ECG)	Diagnostic variables, such as ECG/EEG
Medicine intake (diuretics, inotropics, vasodilators)	Medicine/drug intake
Readmission details (time of readmission, emergency readmissions)	Readmission to hospital

CHF, congestive heart failure; EEG, electroencephalogram.

The table compares variables generated by ML models PhysioNet and HCUP.

### 3.7 Ranking of HF patients

Higher-risk patients were ranked using logistic regression, SVM, and random forest models. Functions named Predict Proba and Log Loss score were used to find the probability of HF patients dead within 6 months, and based on the chances of HF patients dying, the ranking of HF patients was generated with high-risk patients on the top and medium- to lower-risk patients at the bottom.

These rankings were based on the inpatient number (patient ID), a unique value for the HF patient. Refer to Table 6 for the top five ranking of HF patients using logistic regression. These ranked patients were categorized into three categories of importance: first, with <50% probability of mortality; second, with 50%–75% probability of mortality; and last, with 75%–100% probability of mortality. Healthcare organizations can use these categories to provide the required resources, focus, and actions for patients with higher chances of mortality.

**Table 6:** Ranking of patients with heart failure—admissions.

Patient record	Patient ID	Chances of mortality
35	766383	74.06%
450	821102	68.42%
651	823579	67.59%
1,186	797195	63.66%
1,204	729580	62.94%

The table shows the ranking of high-risk HF patients generated by Logistic Regression model.

### 3.8 Validation with CTAS triage model

The final step to add more credibility to research outcomes is to validate the results of PhysioNet data with the CTAS triage model. The first key CTAS triage variable takes a critical look at HF patients to check airways, breathing, circulation, and disability, which connects with demographic variables for current research. The second CTAS variable is a subjective assessment carried out by speaking with the patient, which connects to this research's prognosis, comorbidity, and medicine intake parameters. The third CTAS variable identifies key patient complaints, connected to checking clinical variables for this research. The fourth CTAS variable, objective assessment, connects to the HF patient’s diagnostic and readmission history variables.

## 4 Discussion

Current automated machine learning models for patient prioritization/triage (35) are ineffective enough for the correct selection/classification of HF patients as they miss comorbidity, age, prior patient admission details, and CCI. Implementing automated HF patient prioritization models along with CVD will improve healthcare processes and reduce the cost burden on healthcare.

Prioritization offers healthcare services to the right set of patients in a timely and effective manner, creates a fairness mechanism, and decreases urgent waiting times. It also reduces the unbalanced effects of the differences between patient areas (hospital, ED, or telehealth patients) because it efficiently assigns available resources within each region (36). Automated ML models with patient prioritization logic are excellent decision-support tools and can work for HF patients in emergency settings (37), hospitalization, and remote patient prioritization (38, 39). For HF patients, once hospitalized, comorbidities may not receive the same attention as the primary admitting diagnosis. Currently, most patient prioritization models and wellness strategies for CVD or heart failure patients are based on heart problems as the primary condition, leading to limited and biased outcomes (40).

PP research aims to identify patients with a higher risk of HF during hospital admissions [using the decision-support Patient Prioritization Tool (PPT) tool for health staff]. The high-level research objective includes ranking high- to low-risk patients and understanding the parameters to be checked for HF patients during admissions using exploratory/confirmatory, ML modeling, descriptive analytics, and causal analysis. This research work has brought three critical improvements mentioned below:

1. First improvement: current models used for prioritization during patient admission contain fewer input variables for HF patients. There have been several patient prioritization (triage) models [such as Sakanushi et al. (41), Salman et al. (42, 43), Mohammed et al. (39), Kalid et al. (36), and CTAS (44)] proposed for risk stratification, ranging from schemes based on a few assessments (such as systolic blood pressure and oxygen saturation on admission, blood pressure, and age) to complex models involving multiple factors, such as the development of heart failure, including high blood pressure, hyperlipidemia (high levels of fat in the blood), atherosclerosis, diabetes, obesity, physical inactivity, kidney disease, excessive alcohol intake, and smoking. However, none of these is sufficiently well developed for widespread adoption, mainly because of the variation in presentation and underlying causes limited to acute heart failure (45). This research works on the limitation of earlier studies by increasing the number of critical variables that can be controlled by the healthcare staff while prioritizing patients with HF during hospital admissions.

2. Second improvement: one of the key achievements of this research is the ability to be applied and used in various simple to complex healthcare systems. Across the world, patients with HF receive different services based on the availability of multiple diagnostic tools, machines, hospitals, health facilities, and knowledge of healthcare staff to care for HF patients at admission. Five key buckets (key HF patient variables) identified in this research using two datasets (PhysioNet and HCUP) would provide health staff with a broad set of variables that can be very easy to apply based on the type of healthcare system and geography (villages, small towns, or big cities).

3. Third improvement: the research results went through multiple checks and balances. The six-step process uses exploratory analyses, statistical techniques, ML modeling, Bayesian regression modeling, result validation with the US healthcare system (48,000 records), and cross-checking the results with the CTAS patient prioritization model. These six-step processes were not rigorously followed in any of the past research for HF patients, making the outcome of this research more effective to apply in various healthcare systems and geographies worldwide.

### 4.1 Limitations

The present study has some limitations. The first is that some of the data variables for the PhysioNet dataset were unavailable, which posed the limitation of getting similar types of data after applying the imputation techniques. The second limitation is specific to patients who can be recognized as HF cases at the time of admission. If health staff cannot identify the HF cases, there may be an overlap between heart failure, heart attack, and other heart problems. The third limitation is that the highest risk does not equal the highest benefit during intervention. In several cases, intervention can result in higher benefits in a lower-risk environment, while some patients with very high mortality risk can resist any intervention. The fourth limitation of this study is that the research data reflect the diagnosis of suspected HF patients with high specificity but underestimate the disease burden. The final limitation is applying HF patient prioritization ranking during admission at urgent care units, as there would be a need to apply prioritization for non-HF patients separately to deliver a complete triage process (46) for urgent patients. In summary, the outcome generated from this research can be leveraged for futuristic healthcare areas beyond health operations applied by AI-based prioritizing and ranking critical HF patients for triage (47) and emergency treatments (46). These areas could be virtual patient care, centralized patient admissions for multiple hospitals, centralized wellness engagements using healthcare providers, population health management, and patient self-management of care.

## Data Availability

The original contributions presented in the study are included in the article/**Supplementary material**, further inquiries can be directed to the corresponding author.
