# Supplementary material for: Admission prioritization of heart failure patients with multiple comorbidities
Source: Front Digit Health. 2024 Jul 2;6:1379336. doi: 10.3389/fdgth.2024.1379336 (PMC11250659; doi:10.3389/fdgth.2024.1379336)
Supplement: Supplementary file 1 [file Table1.docx]

Supplementary Material

# Supplementary Figures and Tables

**Table S1**. Classification/prognosis parameters of HF patients- Admission

| **Key Parameters** | **Died in 6 months** | **Not dead in 6 months** | **Total Cases** |
| --- | --- | --- | --- |
| GCS (Avg) | 12.4 | 14.9 | NA |
| NYHA-II | 3% | 97% | 18% |
| NYHA-III | 2% | 98% | 52% |
| NYHA-IV | 5% | 95% | 31% |
| Killip-I | 1% | 99% | 26% |
| Killip-II | 2% | 98% | 51% |
| Killip-III | 5% | 95% | 20% |
| Killip-IV | 27% | 73% | 3% |
| CCI (Avg) | 1.96 | 1.85 | NA |

Note. The above Table shows the average and percentage comparison of HF patients' prognosis parameters to determine their condition at admission.

**Table S2**. Diagnostic/demographic/clinical parameters data for HF patients- admission

| **Key Parameters** | **Died in 6 months** | **Not dead in 6 months** | **Total Cases** |
| --- | --- | --- | --- |
| Respiratory Support (required) | 19% | 81% | 2% |
| LVEF (Avg) | 51.00 | 50.40 | 50.60 |
| Mitral Valve opening (Avg) | 0.926/0.93 | 4.41/4.15 | 4.84/4.0 |
| Blood Pressure- Diastolic (Avg) | 72.00 | 77.00 | 76.57 |
| Blood Pressure- Systolic (Avg) | 126.00 | 131.00 | 131.00 |
| BMI (Avg) | 21.00 | 21.80 | 21.70 |

Note. The above Table compares the diagnostic parameters of HF patients at the time of admission of patients who died vs. those who lived vs. total patients.

**Table S3**. Medicine Intake parameters of HF patients- Admission

| **Key Parameters** | **Died in 6 months** | **Not dead in 6 months** | **Total Cases** |
| --- | --- | --- | --- |
| Diuretic | 3% | 97% | 99% |
| Inotropic | 3% | 97% | 89% |
| Vasodilator | 2% | 98% | 78% |

Note. The above Table compares the medicine intake of HF patients who died vs. those who lived vs. total patients in the study.

**Table S4**. Readmission variable for HF Patients- Admission

| **Key Parameters** | **Died in 6 months** | **Not dead in 6 months** | **Total Cases** |
| --- | --- | --- | --- |
| Readmission 28 days | 0% | 100% | 7% |
| Readmission 3 months | 0% | 100% | 25% |
| Readmission 6 months emergency | 0% | 100% | 39% |

Note. The above Table compares readmission data for HF patients who died vs. who lived vs. total patients in the study.

**Table S5**. Comorbidity percentage comparison for HF patients- Admission

| **Key Parameters** | **Died in 6 months** | **Not dead in 6 months** | **Total Cases** |
| --- | --- | --- | --- |
| Liver disease | 11% | 89% | 4% |
| CKD | 5% | 95% | 24% |
| Renal Failure | 29% | 71% | 0% |
| Diabetes | 3% | 97% | 23% |
| Dementia | 1% | 99% | 6% |
| COPD | 2% | 98% | 12% |
| CHF | 2% | 98% | 93% |

Note. The above Table shows the percentage comparison of HF patients suffering from comorbidities who died vs. who lived vs. total cases after their admission to the hospital.

**Table S6**. Logistic regression model key indicators in descending order- Admission

| **Feature** | **Feature importance** |
| --- | --- |
| Killip grade | 1.626472 |
| Return to emergency department within 6 months | 1.271204 |
| Readmission within 3 months | 0.806962 |
| Chronic kidney disease | 0.544461 |
| Liver disease | 0.476219 |
| Type of heart failure | 0.422968 |
| Eye-opening | 0.398072 |
| consciousness | 0.341475 |
| Body temperature | 0.334738 |
| Respiratory support | 0.228956 |
| Vasodilators | 0.227165 |
| Readmission within 28 days | 0.223194 |
| movement | 0.204775 |
| NYHA cardiac function classification | 0.195403 |
| Inotropic | 0.187695 |
| Acute renal failure | 0.18038 |
| Age Cat | 0.178745 |
| dementia | 0.17044 |
| respiration | 0.166106 |
| Chronic pulmonary disease | 0.152291 |
| CCI score | 0.120799 |

Note. The above Table shows the variables coming from logistic regression- Admission.

**Table S7**. Support vector machine (SVM) model key indicators in descending order

| **Feature** | **Feature Importance** |
| --- | --- |
| Eye-opening | 5.17E-01 |
| Return to emergency department within 6 months | 2.59E-01 |
| Respiratory support | 2.35E-01 |
| GCS | 1.79E-01 |
| movement | 1.71E-01 |
| Verbal response | 1.67E-01 |
| Readmission within 3 months | 1.59E-01 |
| AIDS | 1.00E-01 |
| consciousness | 1.00E-01 |
| Liver disease | 7.37E-02 |
| dementia | 5.89E-02 |
| Readmission within 28 days | 5.89E-02 |
| Chronic kidney disease | 5.03E-02 |
| Acute renal failure | 3.34E-02 |
| Peptic ulcer disease | 3.20E-02 |
| Killip grade | 2.67E-02 |
| CCI score | 2.47E-02 |
| Myocardial infarction | 1.83E-02 |
| Chronic pulmonary disease | 1.52E-02 |
| Inotropic | 1.51E-02 |
| diabetes | 1.31E-02 |
| Mitral valve AMS | 1.30E-02 |
| gender | 1.16E-02 |
| Solid tumor | 8.40E-03 |
| Type of heart failure | 5.35E-03 |

Note. The above Table shows the variables coming from SVM- Admission.

**Table S8**. Random forest model key indicators in descending order- Admission

| **Feature** | **Feature Importance** |
| --- | --- |
| consciousness | 1.03E-01 |
| Eye-opening | 1.01E-01 |
| GCS | 8.79E-02 |
| Verbal response | 7.00E-02 |
| Killip grade | 6.33E-02 |
| Diastolic blood pressure | 5.68E-02 |
| pulse | 5.57E-02 |
| Systolic blood pressure | 3.96E-02 |
| Oxygen saturation | 3.79E-02 |
| CCI score | 3.79E-02 |
| LVEF | 3.64E-02 |
| Mitral valve AMS | 3.57E-02 |
| Body temperature | 3.00E-02 |
| BMI | 2.91E-02 |
| Mitral valve EMS | 2.77E-02 |
| respiration | 2.64E-02 |
| movement | 2.27E-02 |
| Acute renal failure | 2.22E-02 |
| Chronic kidney disease | 1.59E-02 |
| Return to emergency department within 6 months | 1.53E-02 |

Note. The above Table shows the variables coming from Random Forest- Admission.

**Table S9**. Linear Regression (binomial/Bayesian regression) outcome- Admissions

| Bayesian Parameters | Key Parameter 1 | Key Parameter 2 | Key Parameter 3 |
| --- | --- | --- | --- |
| Classification | βGCS = −0.291601 | βNYHA = −0.121965 | βCCI = −0.028992 |
| Diagnostic | βsystolicbp = −0.001597 | βdiastolicbp = −0.02143 | βECGAMS = −0.022431 |
| Medicinal Data | βdiuretics = 14.149534 | βinotropics = 0.262056 | βvasodilators = −0.603034 |
| Readmission | βreadmission3months = −9.732389 | βreturnED6months = −72.483767 |  |
| Comorbidity | βliverdisease = 1.48249 | βCKD = 0.8753 | βCOPD = −0.59304 |

**Table S10**. Logistic regression model for state data of Maryland- Admissions

| **Feature** | **Feature Importance** |
| --- | --- |
| PHYTHERAPY | 1.62E+00 |
| ED | 1.07E+00 |
| CHRON3 | 7.90E-01 |
| RESPTHERAPY | 7.85E-01 |
| CHRON2 | 6.91E-01 |
| OBESE | 5.59E-01 |
| COAG | 4.16E-01 |
| EEG | 4.02E-01 |
| OBSERVATION | 3.99E-01 |
| HTN | 3.89E-01 |
| DRUG | 3.85E-01 |
| CHRON1 | 3.57E-01 |
| READMIT | 3.53E-01 |
| ICU | 3.49E-01 |
| WGHTLOSS | 3.38E-01 |
| DEPRESS | 3.03E-01 |
| EKG | 2.98E-01 |
| DMCX | 2.82E-01 |
| METS | 2.61E-01 |
| PSYCH | 2.47E-01 |
| VALVE | 2.45E-01 |
| CHRNLUNG | 2.42E-01 |
| CCU | 2.32E-01 |
| ANEMDEF | 2.21E-01 |
| PARA | 2.05E-01 |
| RENLFAIL | 1.97E-01 |

Note. The above Table shows the variables from logistic regression- Admission- HCUP.


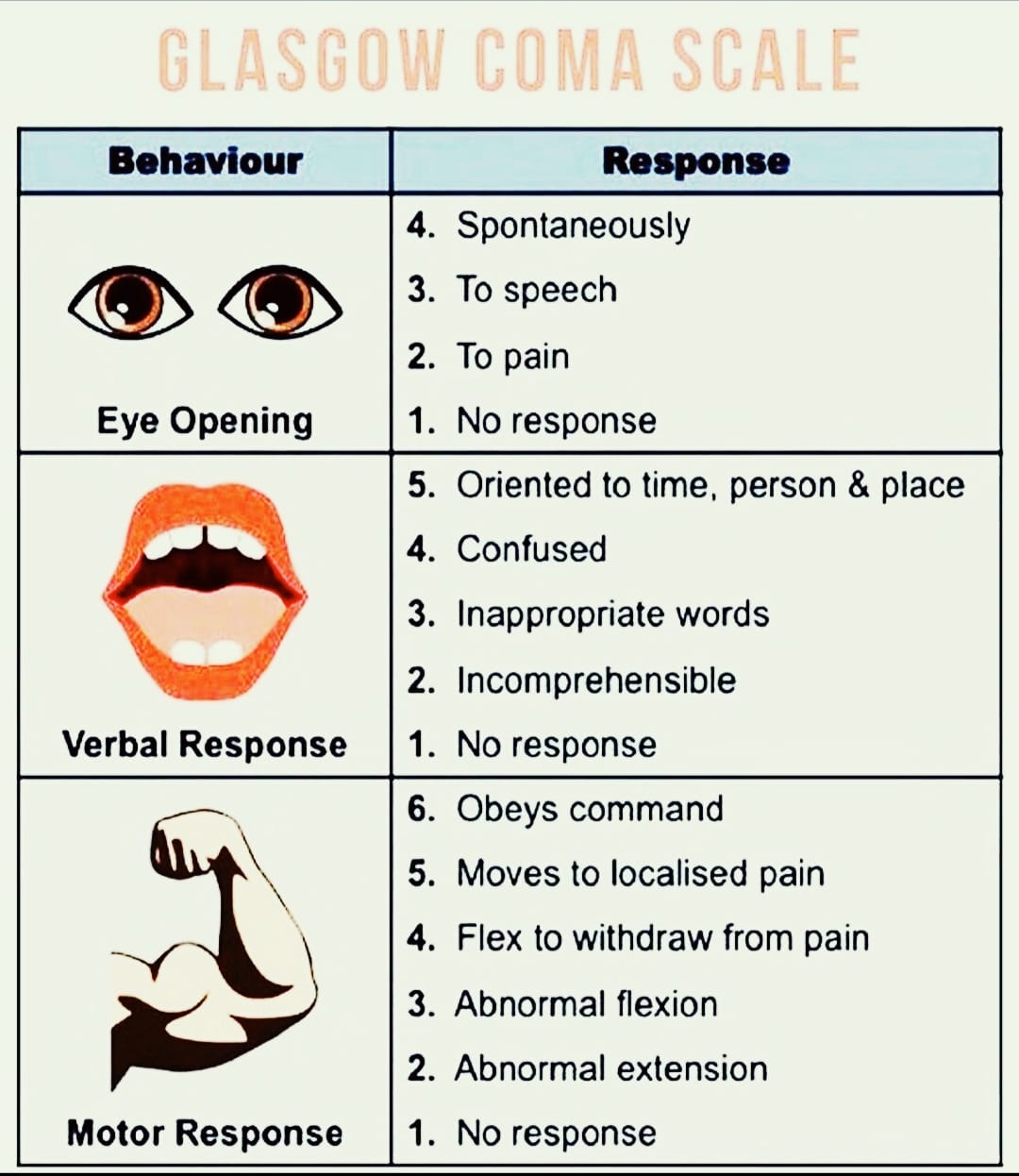


**Figure S1**. Glasgow Coma Scale (GCS). GCS is an excellent observational index used to identify the patient's condition (Eye/verbal/motor functions)

**Figure S2**. New York Heart Association (NYHA). NYHA helps in the easy classification of HF patients based on observations

**Figure S3**. Killip Grade. Killip Grade is a prognosis factor to identify the Heart condition of a patient and is divided into 1 to 4 grades

**Figure S4**. Charlson Comorbidity Index (CCI). Classification of patients based on chronic comorbidity conditions

***
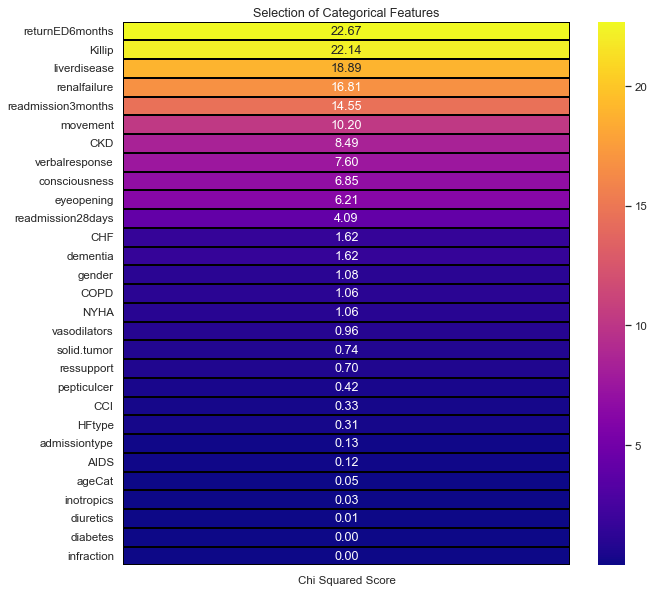
***

**Figure S5**. Chi-square test for categorical variables-PhysioNet. The chi-square test indicates variables of higher importance for Categorical data.


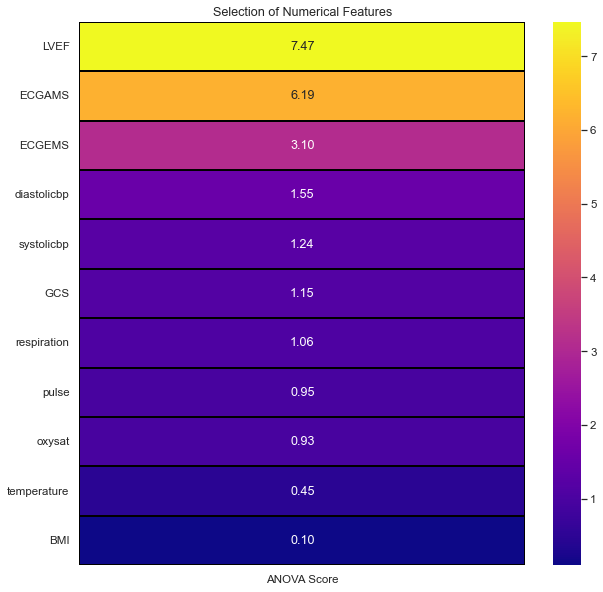


**Figure S6**. ANOVA test for continuous Variable-PhysioNet. ANOVA test indicates key variables of higher importance for numerical data.

**
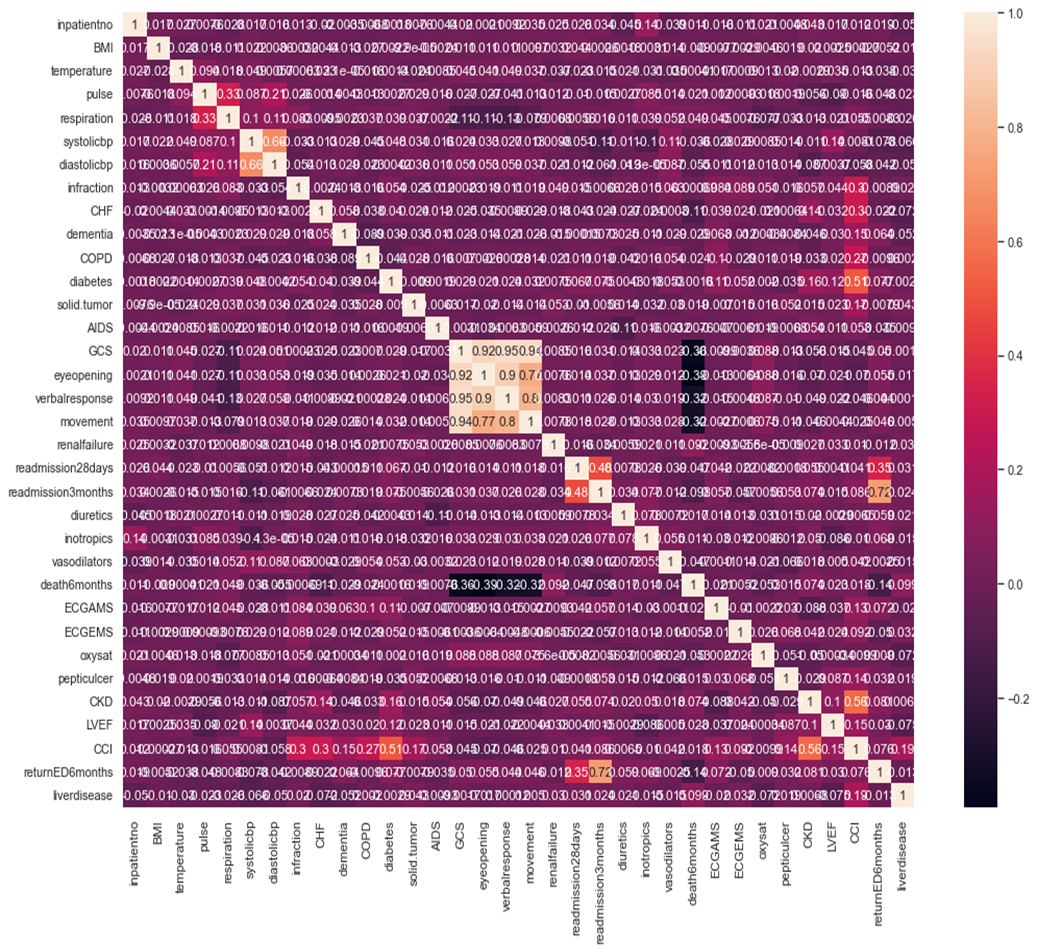
**

**Figure S7**. Correlation variables- Admission-PhysioNet. Correlation chat indicates the input variables with a high correlation value

**
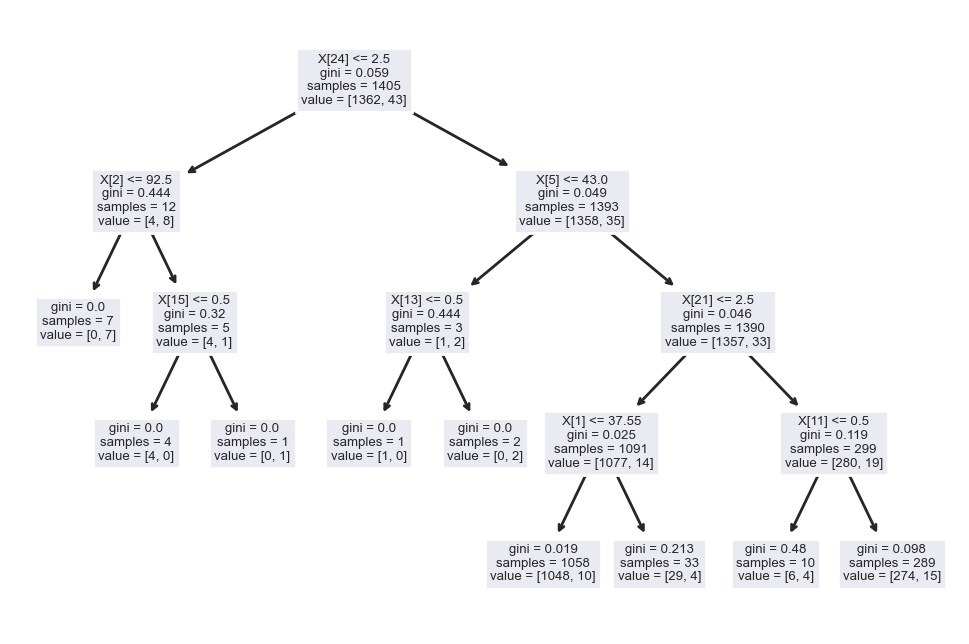
**

**Figure S8**. Decision Tree with decision nodes Classification and Diagnosis variables- PhysioNet. The decision Tree chart indicates the key variables used to divide and segregate the data variables.


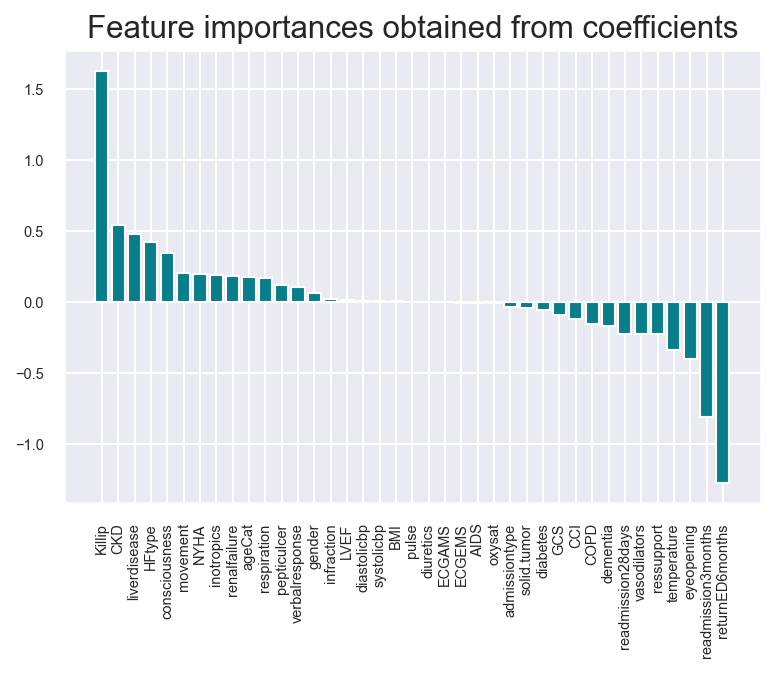


**Figure S9**. Condition variables of HF patients at the time of admission- all variables (logistics)- PhysioNet. The logistic regression chart indicates key input variables at the time of admission for HF patients.

**
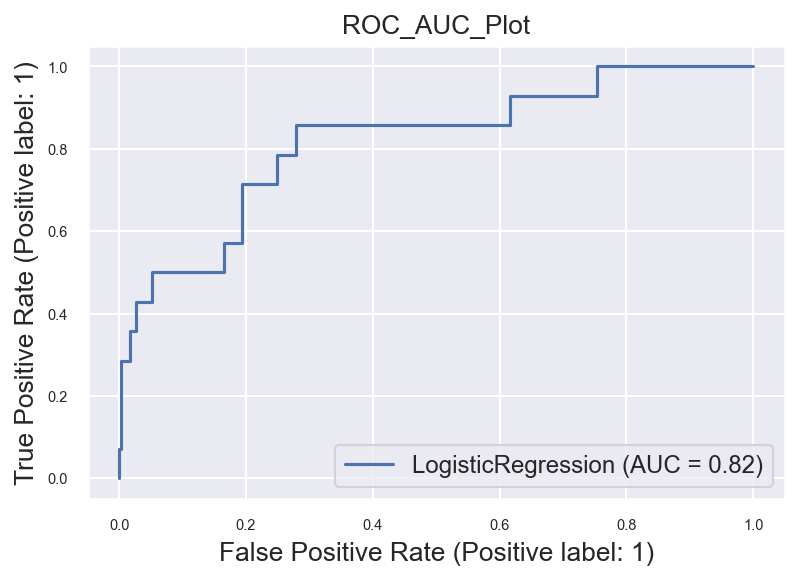
**

**Figure S10**. The area under the curve for HF patients at the time of admission- all variable (logistics)-PhysioNet. Model Accuracy: 97.84%; Cross-Validation Score: 78.29%; ROC_AUC Score: 60.54%.


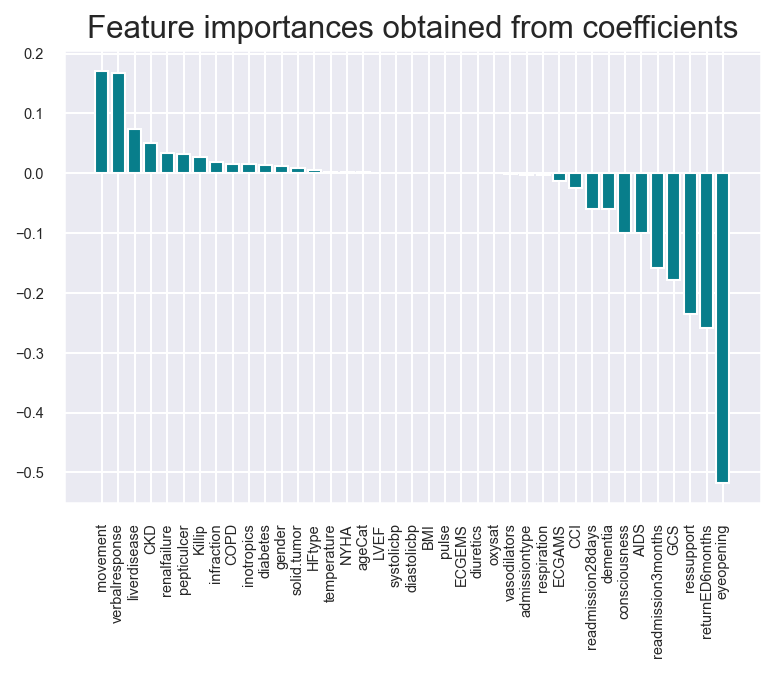


**Figure S11**. Condition variables of HF patients at the time of admission (SVM) all variable- PhysioNet. The SVM chart indicates key input variables of higher importance at the time of admission for HF patients.

**
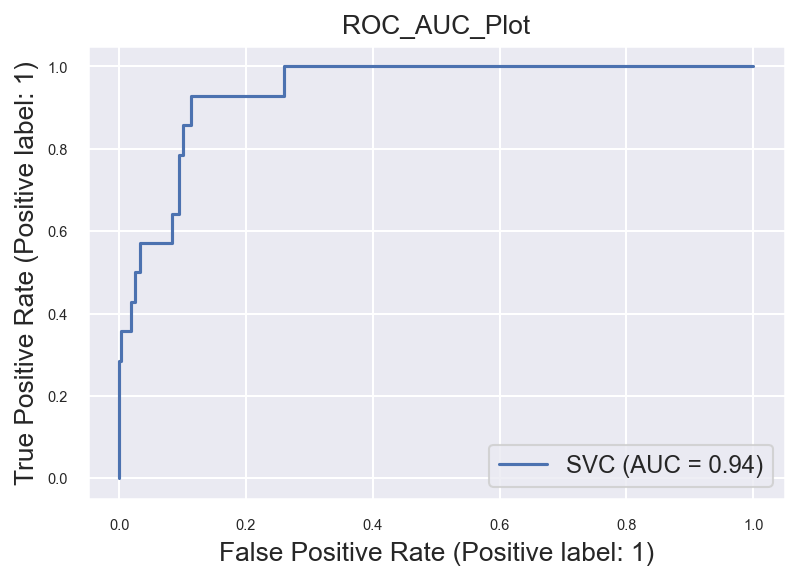
**

**Figure S12**. The area under the curve for HF patients at the time of admission (SVM) all variables- PhysioNet. Accuracy:98.34%; Cross Validation Score:80.20%; ROC_AUC Score:64.29%.

**
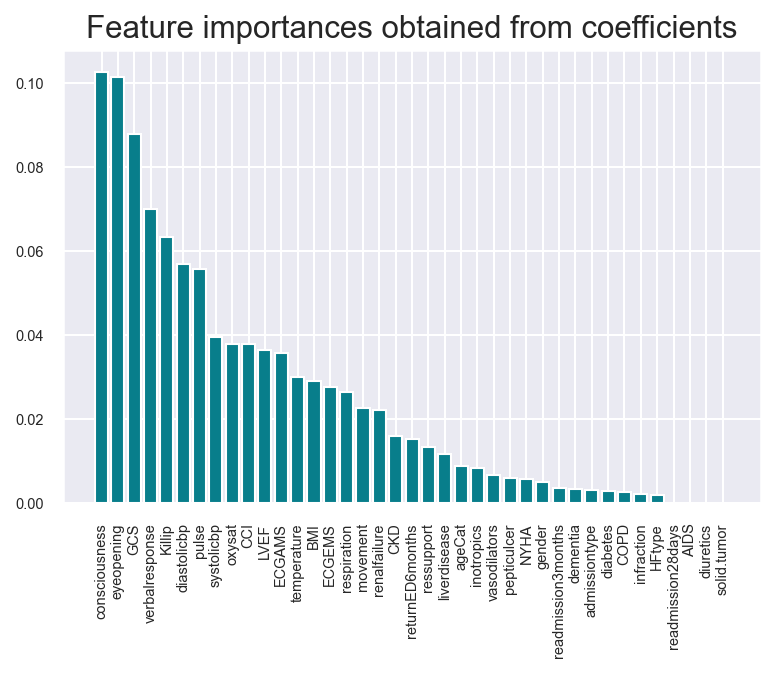
**

**Figure S13**. Condition variables of HF patients at the time of admission (random forest) all variables- PhysioNet. Random Forest chart indicates key input variables of higher importance (without the positive or negative influence of variables) at the time of admission for HF patients.

**
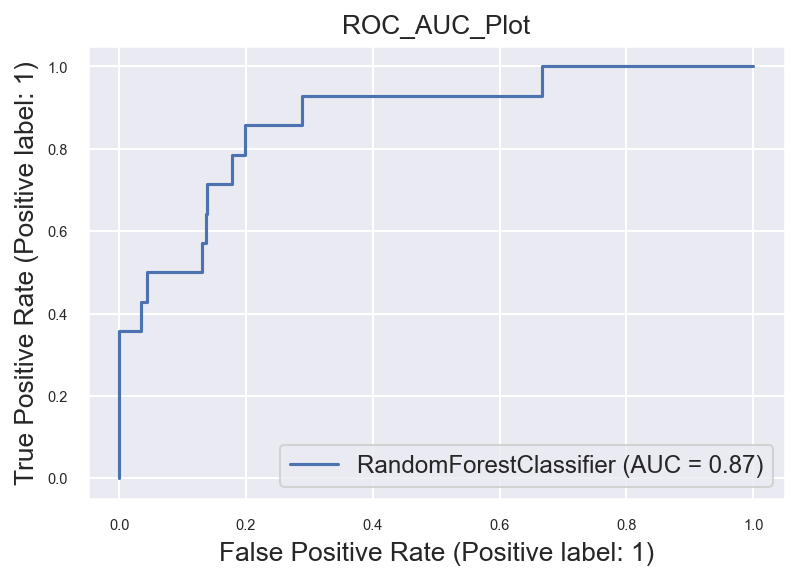
**

**Figure S14**. AUC curve of HF patients at the time of admission all variables (random forest)- PhysioNet. Accuracy: 98.34%; Cross-Validation Score: 82.86%; ROC_AUC Score: 64.29%.


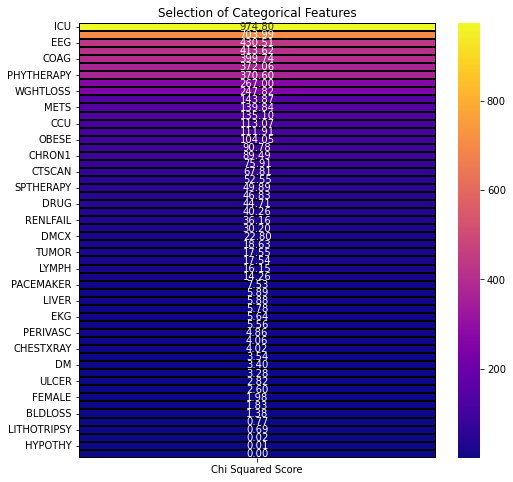


**Figure S15**. Chi-Square test for state data- MD 2014 (HCUP). Key parameters are comorbidities, such as Liver disease, Diabetes, Renal failure, and Tumor/cancer; Diagnostic variables, such as EEG, EKG, Obesity, and therapy; patient classification variables, such as Chron1 (part of Chronic illness, CCI) and medicine/drug intake.

*
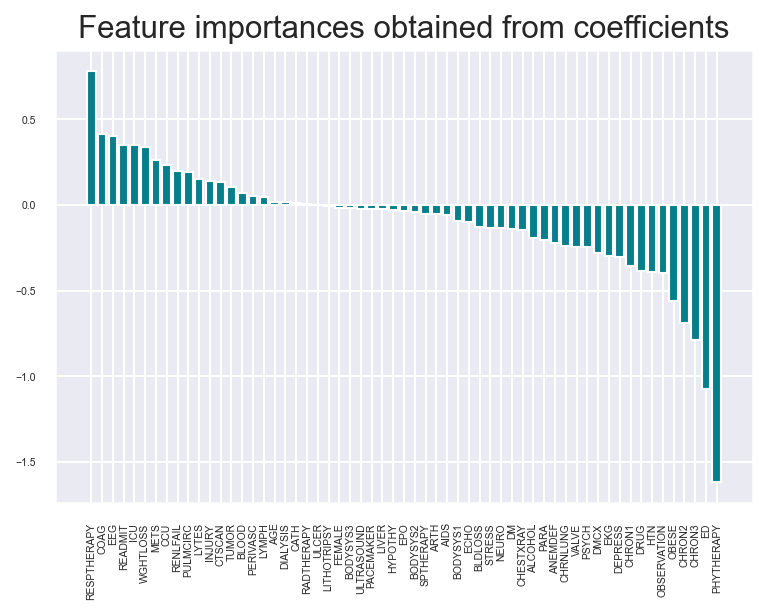
*

**Figure S16**. Logistic regression for state data- MD 2014. The above figure shows the importance of HCUP data variables with a logistic regression model.

**
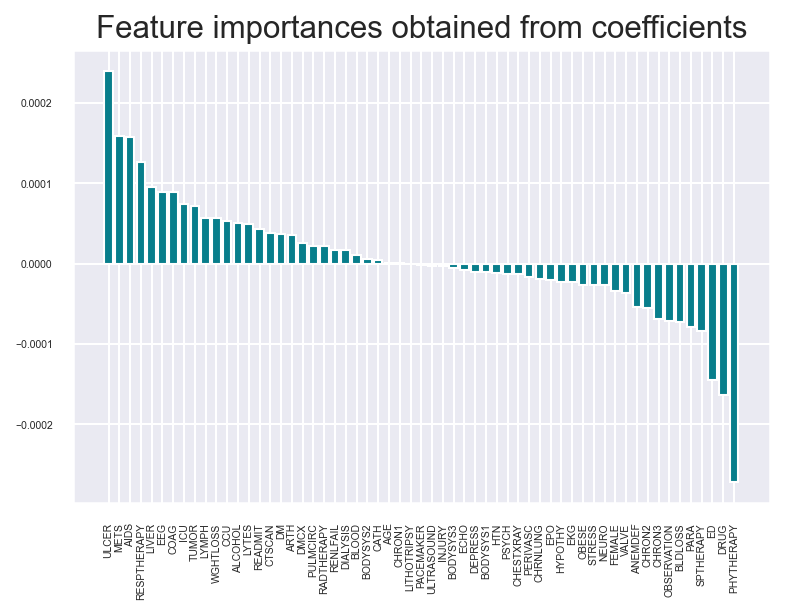
**

**Figure S17**. Support Vector Machine regression for state data- MD 2014. The above figure shows the importance of HCUP data variables with the SVM regression model, and it is coming out to be in similar lines with the primary research generated with the PhysioNet dataset.

**
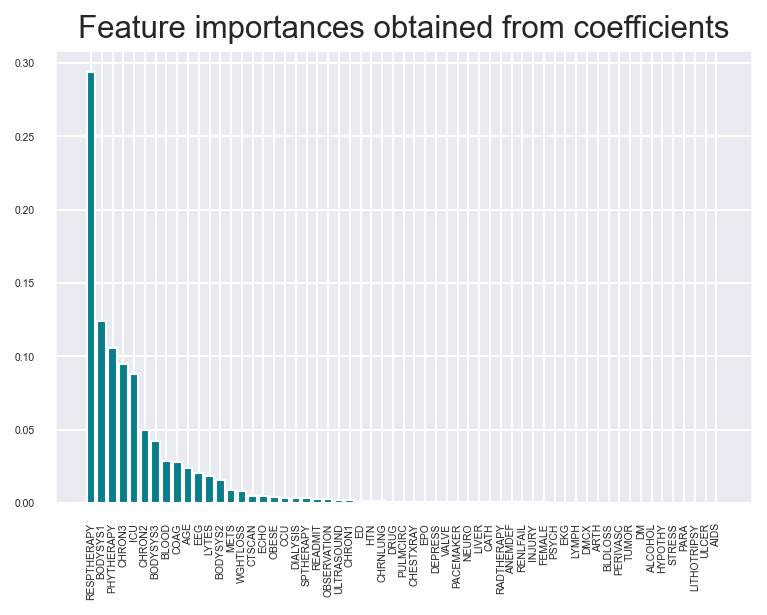
**

**Figure S18**. Random Forest for state data- MD 2014. The above figure shows the importance of HCUP data variables with the Random forest model, and it is coming out to be in similar lines with the primary research generated with the PhysioNet dataset.
